# Multiple object handling: exploring strategies for cumulative grasping and transport using a single hand

**DOI:** 10.1007/s00221-025-07084-x

**Published:** 2025-06-03

**Authors:** Arran T. Reader, Laura Gaile, Wenxi Li, Emily E. Cheah Mc Corry, Kirsten Mackie

**Affiliations:** Department of Psychology, Faculty of Natural Sciences, University of Stirling, Stirling, UK

**Keywords:** Atypical grip, Finger-thumb opposition, Manual dexterity, Object properties

## Abstract

Humans can cumulatively grasp multiple objects and then transport them using a single hand (e.g., when clearing up tableware). This skill, which we refer to as ‘multiple object handling’, helps minimise the number of actions required to transport objects. However, it also presents unique challenges for the sensorimotor system, including the use of grips other than finger-thumb opposition (almost always used for grasping single objects). In the present work we explored the strategies used for multiple object handling, particularly focussing on object selection and grip choice. Participants were presented with pairs of objects, asked to grasp one of them and then, without placing that object down, grasp the second object and transport both to a designated location. We examined the order in which participants selected objects and the grips used for grasping and holding them. Results provide preliminary evidence for a typical approach to multiple object handling. We observed that when two objects were grasped cumulatively for transport, finger-thumb opposition was almost always used to grasp the first object, which was then frequently held using an atypical grip (e.g., finger-finger or finger-palm opposition). Finger-thumb opposition was almost always used once again to grasp the subsequent object. Participants preferred to grasp objects with lower mass or surface area first, potentially facilitating this approach. In sum, this work provides insight into a technique commonly used for efficient object transport.

**Supplementary Information:**

The online version contains supplementary material available at 10.1007/s00221-025-07084-x.

## Introduction

Human manual dexterity is highly refined thanks to an extensive network for motor control distributed across the brain, as well as the structure of the hand and our ability to synergistically control its many component parts (Errante et al. 2021; Sobinov and Bensmaia 2021; Yan et al. 2020). Our versatility in manual control is not only highlighted by the range of actions we can perform (e.g., tool use, gesturing, instrument playing), but also in our daily use of common objects, where we are easily able to manipulate handheld items regardless of their shape, size, or texture.

Grasping a single object in preparation for transport typically involves the opposition of the palmar surfaces of the fingers and the thumb (Castiello 2005). For example, larger objects often elicit a ‘power’ grip, where all fingers oppose the thumb to encompass the object. Conversely, smaller objects are more likely to elicit a ‘precision’ grip, with objects being held between the thumb and the index (and often middle) finger. ‘Grasp taxonomies’ (e.g., Feix et al. 2016; Stival et al. 2019) have shown that whilst a variety of grips might be used during object interaction, they almost always involve finger-thumb opposition in some form (suitable for the target object), even if non-palmar surfaces are occasionally involved in the grip (Gonzalez et al. 2014). Unsurprisingly then, finger-thumb opposition has been extensively studied for several decades, revealing in detail its sensitivity to object properties and how these can influence the spatial and temporal features of hand shaping (such as the extent and timing of hand opening, e.g., Ansuini et al. 2015; Betti et al. 2018; Castiello et al. 1993; Cuijpers et al. 2004; Egmose and Køppe 2018; Gentilucci et al. 1991; Jeannerod 1984; Marteniuk et al. 1990; Paulun et al. 2016; Savelsbergh et al. 1996; Weir et al. 1991). Such work has helped generate a comprehensive understanding of human object interaction.

**Fig. 1:**
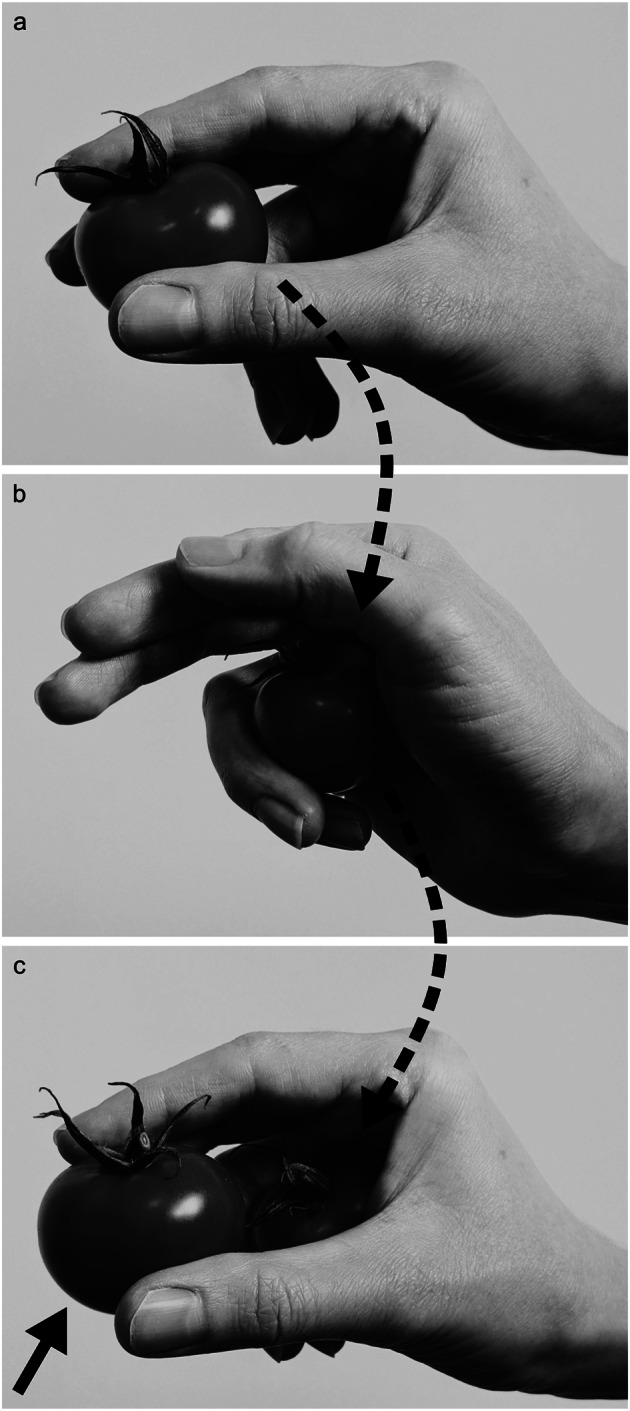
An example of multiple object handling. (**a**) When selecting tomatoes at the supermarket, we might typically grasp one between the thumb, index, and middle fingers. (**b**) If we wished to select further tomatoes before transporting them, we could secure the first between the palm, ring, and little fingers (first dashed line). (**c**) With the first tomato remaining in the hand (second dashed line), another one can be grasped (solid line). It would also be possible to hold more than two tomatoes in the hand prior to transport, depending on their size (not shown)

Nevertheless, our daily interactions with objects can be more complex than reaching to and grasping them individually. In fact, we frequently grasp multiple objects in a cumulative fashion and then transport them with a single hand. For example, at the supermarket, we might select several tomatoes one-by-one with the right hand before placing them all into a bag held in our left (Fig. 1). If we’re heading to a meeting at work, we can first take our pen and then our notebook in one hand, leaving the other hand free for our coffee cup. This ability, which we refer to as ‘multiple object handling’, provides an efficient way of using the hand to minimise the number of actions required to complete a goal.

Unlike single-object interactions, multiple object handling can frequently elicit the use of grips other than those involving finger-thumb opposition, which are infrequently studied and rarely considered in published grasp taxonomies. A common example of such an atypical grip can be observed when clearing tableware following a meal: after picking up a drinking glass, we can more quickly complete our task by pinching a second one between the sides of two fingers. In the supermarket example described above, we can first grasp a tomato in a typical manner with the fingers opposing the thumb before securing it in the palm using our ring and little fingers (an atypical grip). This enables us to then grasp a second tomato (Fig. 1). We could also, with relatively little difficulty, use an atypical grip to *grasp* a tomato in this scenario (i.e., opposing the palm with our ring and little fingers to select a second tomato when one is already held between the thumb and adjacent fingers). We are successful in performing such movements despite the apparent challenges they present for the sensorimotor system: atypical grips might involve parts of the hand that have more limited fine control (the sides of the fingers) or independence (the ring and little fingers). In addition, multiple object handling may require rapid parallel and sequential coordination of different parts of the hand to ensure that a previous object is held securely whilst grasping the next one. This appears to be more complex than grasping a single object, and requires effectively planning for the varied properties of multiple objects and how those properties might interact.

It remains unclear how humans engage in multiple object handling to facilitate everyday object interaction. How do we choose which order to select objects, thus ensuring that they can all be transported effectively? How do we grip each object when we grasp or hold it, and how might this relate to the features of the different objects? As a preliminary examination of this behaviour, we sought to explore the strategies used for multiple object handling, particularly focussing on the grip types used for grasping and holding objects (either typical finger-thumb opposition, or atypical). Participants were presented with pairs of familiar objects specifically chosen to elicit a wide variety of grips. In each trial participants were asked to grasp either one of the objects and then, without placing that object down, grasp the second object and transport both to a designated location. By examining the order in which participants selected objects, as well as the grips used for grasping and holding them at different stages of the movement, we could provide insight into how humans engage in multiple object handling.

## Method

### Participants

We recruited 30 participants from the University of Stirling and the surrounding area. Participants were right-handed (self-report) and aged between 18 and 33 years, mean±SD = 21.3±4.54 years, 13 male, 17 female. If participants were undergraduate students studying Psychology modules at the University of Stirling, then they were awarded research tokens which contributed to course requirements. Other participants did not receive any incentive. All participants provided written informed consent and the project was approved by the University of Stirling General University Ethics Panel (approval number: GUEP 2022 8687 7551).

### Materials

To examine multiple object handling in a semi-naturalistic manner we used twelve familiar objects that could elicit a variety of grips. The objects were chosen such that, if grasped individually, they provided opportunities for the majority of finger-thumb opposition grips detailed in recent grasp taxonomies (Feix et al. 2016; Stival et al. 2019). The alignment with these grasp taxonomies is provided in Supplementary Material (Table S1).

Objects were as follows: a drinking glass (diameter of 64 mm, height of 160 mm, 263.7 g), a metal deodorant can (diameter of 50 mm, tapering to 18 mm at the nozzle, height of 200 mm, 35.2 g), a ceramic plate (diameter of 117 mm, height of 20 mm, 123.8 g), a paperback book (228 × 152 × 21 mm, 425.5 g), a tennis ball (diameter of 65 mm, 57.8 g), a plastic icing rolling pin (diameter of 26 mm, length of 150 mm, 77.8 g), a table tennis (or ‘ping pong’) ball (diameter of 40 mm, 2.7 g), an unsharpened wooden pencil with an eraser (diameter of 7 mm, length of 188 mm, 4.8 g), a plastic dice (side lengths of 15 mm, 6.1 g), a metal key (approx. 50 × 22 × 2 mm, 6.2 g), a metal nut (diameter of 6 mm, height of 2 mm, 0.3 g), and a blunt metal sewing needle (length of 52 mm, 0.5 g).

**Fig. 2:**
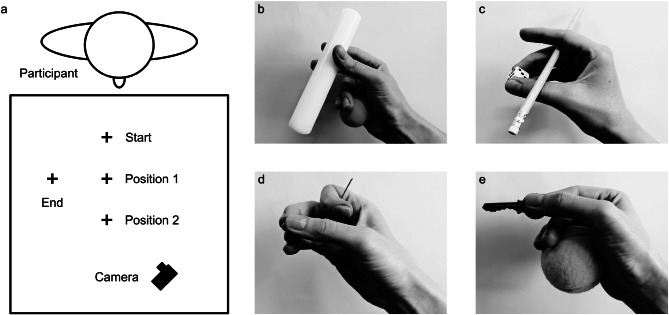
Experimental setup and example grip types shown for transport. (**a**) Experimental setup. (**b**) Object 1: Table tennis ball (*Finger-Palm*), Object 2: Rolling pin (*Finger-Thumb*). (**c**) Object 1: Pencil (*Finger-Finger*), Object 2: Dice (*Finger-Thumb*). (**d**) Object 1: Needle (*Other*), Object 2: Nut (*Finger-Thumb*). Note that this is just one example of the possible grips captured in the *Other* category. (**e**) Object 1: Key (*Finger-Thumb*), Object 2: Tennis ball (*Finger-Palm*). Note the use of an atypical grip for grasping Object 2

Participants were seated on an office chair at a square table with an 80 cm^2^ surface (Fig. 2a). White tape was used to create 4 small crosses on the table. One cross was located 35 cm from the right side of the table, and 15 cm from the edge of the table nearest the participant, indicating the starting position of the participant’s hand. A second cross (Position 1) was located 15 cm beyond the starting position. A third cross (Position 2) was located 15 cm beyond Position 1. A fourth cross was placed 20 cm to the right of Position 1, indicating the location participants were required to move objects to after grasping them. Lastly, a camera was used for video-recording trials, located 30 cm to the left of Position 2, at a 45° angle. The camera used was an AKASO EK7000 action camera (AKASO Tech LLC). Videos were recorded at 4K resolution and at 30 frames-per-second.

### Design and procedure

The twelve objects were presented to each participant four times, twice in Position 1 and twice in Position 2 (with two objects presented in every trial). As such, participants completed 24 trials. Object combinations were randomised aside from the requirement than no replication of object and position combination occurred for any one participant. Randomisation was performed in Microsoft Excel. Each object and position combination appeared at least once in the sample, with a range of 1–12 trials, mean ± SD = 5.45 ± 2.41 trials.

Participants were provided with a demonstration of the task by the experimenter before data collection began, using the first object pair presented to that participant. Participants began each trial with their right index finger resting in the centre of the starting position. One object was placed on Position 1 and one object on Position 2. The key, pencil, and sewing needle were placed in an ‘action’ position (i.e., placed with the typically grasped end pointing towards the participant’s right hand). The deodorant can and drinking glass were placed upright whilst the rolling pin was placed flat on the table, in a horizontal orientation relative to the participant.

Participants were asked to grasp one object and then, without letting that object touch the table again, grasp the second object and move both objects to the end position. Participants could grasp the objects in any order and place them at the end position however they wished. They then returned their hand to the start position. It was made clear to participants that they could grasp the objects in any manner they chose, however they were asked to repeat any trials if they let the first object touch the table after lifting it or attempted to use one object to carry another instead of grasping both with their hand. After each trial the objects were immediately replaced for the following trial.

### Data analysis

#### Coding

For each trial, we recorded the order in which objects were selected (designating these Object 1, Object 2). Three participant videos were used for developing a coding system, and these participants (1, 11, and 21) were excluded from further analysis. The coding system aimed to capture the grip types used by participants when grasping or holding each object. Specifically, we categorised how objects were gripped based on the opposing effectors used, examining both typical (*Finger-Thumb*), and atypical (*Finger-Finger*, *Finger-Palm*) grips. *Finger-Finger* grips were considered to be cases in which an object was gripped between two or more fingers. *Finger-Palm* grips were those in which an object was held within the palm using the fingers. Categorisation was based on the effectors which were primarily used to secure the object, and not all parts of the hand in contact with the object. For example, a power grip was categorised as *Finger-Thumb*, even if the object was in contact with the palm, because the fingers and the thumb are the opposing effectors. We also included a fourth category (*Other*), for atypical grips which did not meet the above criteria. For example, cases in which the thumb opposed the palm, fingers opposed themselves (i.e., an object was ‘hooked’ within the fingers or at the palmar digital crease), or the palm opposed itself (i.e., when a small object was held within the distal or proximal palmar crease).

We coded the grip for three stages of the action sequence in each trial. The first two stages were Object 1 lift (the grip used to lift Object 1) and Object 1 hold (the grip used to secure Object 1 at the point of grasping Object 2). The third stage was Object 2 lift (the grip used to lift Object 2). Examples of each grip type are shown for pairs of objects as if for transport in Fig. 2. Trials were excluded from further analysis if objects were placed in the wrong positions by the experimenters, the incorrect objects were placed, or in the case of one participant who slid some objects to the edge of the table to grasp them rather than lifting them. Based on these criteria, 1.39% of trials were excluded (*n* = 9). Readjustment of the grip for either object was rare and this was not considered during coding. Coding was performed by one author (EEC) and then independently verified by a second (ATR), who resolved any disagreements (agreement = 87.6%).

#### Object selection analysis

We examined which objects were preferentially chosen to be grasped first. Originally, we planned to perform only a descriptive analysis of object order preference. However, following the suggestion of a peer reviewer, we also quantitatively evaluated the possible link between specific object properties and grasp order. Properties considered were mass and surface area, the latter of which was considered a suitable measure of object size given that it captures the area that can be gripped. Surface area was calculated for the ten objects for which it could be readily approximated (glass = 67740 mm^2^, deodorant = 32771 mm^2^, book = 85272 mm^2^, tennis ball = 13273 mm^2^, rolling pin = 13314 mm^2^, table tennis ball = 5027 mm^2^, pencil = 4211 mm^2^, dice = 1350 mm^2^, nut = 114 mm^2^, needle = 165 mm^2^). Two Spearman’s rank correlations were performed between the percentage each object was grasped first and object mass and surface area (note that object mass and surface area were strongly correlated, r_s_(8) = 0.915, *p* < 0.001). In addition, whilst we did not originally plan to evaluate the influence of object position, we also calculated this following a reviewer suggestion.

#### Grip choice analysis

To evaluate the different grips used within action stages, we calculated the frequency of coded trials in which each grip was used in each stage across the entire sample. For each stage a multinomial test was performed to examine whether the proportion of each grip type (*Finger-Thumb*, *Finger-Finger*, *Finger-Palm*, *Other*) differed from a uniform distribution. Given the three multinomial tests performed, a Bonferroni-corrected alpha value of 0.0167 was used to evaluate statistical significance.

Since object pairings were randomly assigned to participants and participants could freely choose the order in which they grasped objects, the prevalence of specific object-order combinations varied across the sample. As such, a skewed distribution of grip types within a stage is likely to indicate an invariant strategy across participants (who are interacting with different objects). However, to evaluate whether grip distribution might be consistent across participants in specific object-order combinations, we also performed a post hoc analysis on the three cases where over half of the participants (*n* ≥ 14) happened to grasp the same objects in the same order. Multinomial tests were used to examine the proportion of each grip type in each stage for the object-order combinations of pencil (Object 1) and book (Object 2), rolling pin and plate, and table tennis ball and plate. If one of these object-order combinations appeared more than once for a participant, we maintained only the first trial for analysis. A Bonferroni-corrected alpha value of 0.0167 was also used for each of these analyses.

Given that the multinomial tests performed on the whole sample are only (partially) informative regarding the distribution of grip types within stages, Wilcoxon signed-rank tests were used to compare the percentage of each grip type between stages. As with the multinomial tests, whilst all participants have a percentage value for each grip type that can be compared across stages, this is calculated based on different object-order combinations. That is, percentages for each participant are based solely on the objects they were presented with (which is a subset of all possible object-position combinations) and the order in which they chose to grasp them. As such, a large difference in percentage grip type between stages is more likely to reflect an action strategy that is consistent across participants (and less likely to be explained by differences in object pairings). Since each grip type was analysed twice, a Bonferroni-corrected alpha value of 0.025 was used for these analyses. Effect size was calculated as rank biserial correlation.

Note that mean percentage values for each grip and stage can differ slightly between multinomial and Wilcoxon analyses, since for the former analysis frequencies are calculated for the entire sample, whereas in the latter percentages are calculated per participant based on the sum of their non-excluded trials (with some participants having less than 24 trials available for analysis). Statistical tests were performed using JASP (JASP Team 2024) and Jamovi (The Jamovi Project 2024).

To complement the aforementioned statistical analysis, pie charts were created to provide a descriptive analysis and visualisation of the interaction between object selection order and grip choice (for example, to examine which grips were used in each stage when the needle was chosen as Object 1 and the pencil as Object 2). Whilst all objects were chosen at least 10 times as Object 1 (range = 10–89, mean ± SD = 53.3 ± 28.2 trials) or Object 2 (range = 16–97, mean ± SD = 53.3 ± 28.6 trials), some specific object combinations had very few trials (e.g., participants rarely chose the glass as the first object to grasp in a pair). Therefore, we limited visualisation to cases where there were at least 4 trials for an object order combination across all participants (i.e., it was possible for all 4 grip types to occur across the sample). As such, this analysis included 87.2% of non-excluded trials (557 of 639).

## Results

### Object selection

**Fig. 3:**
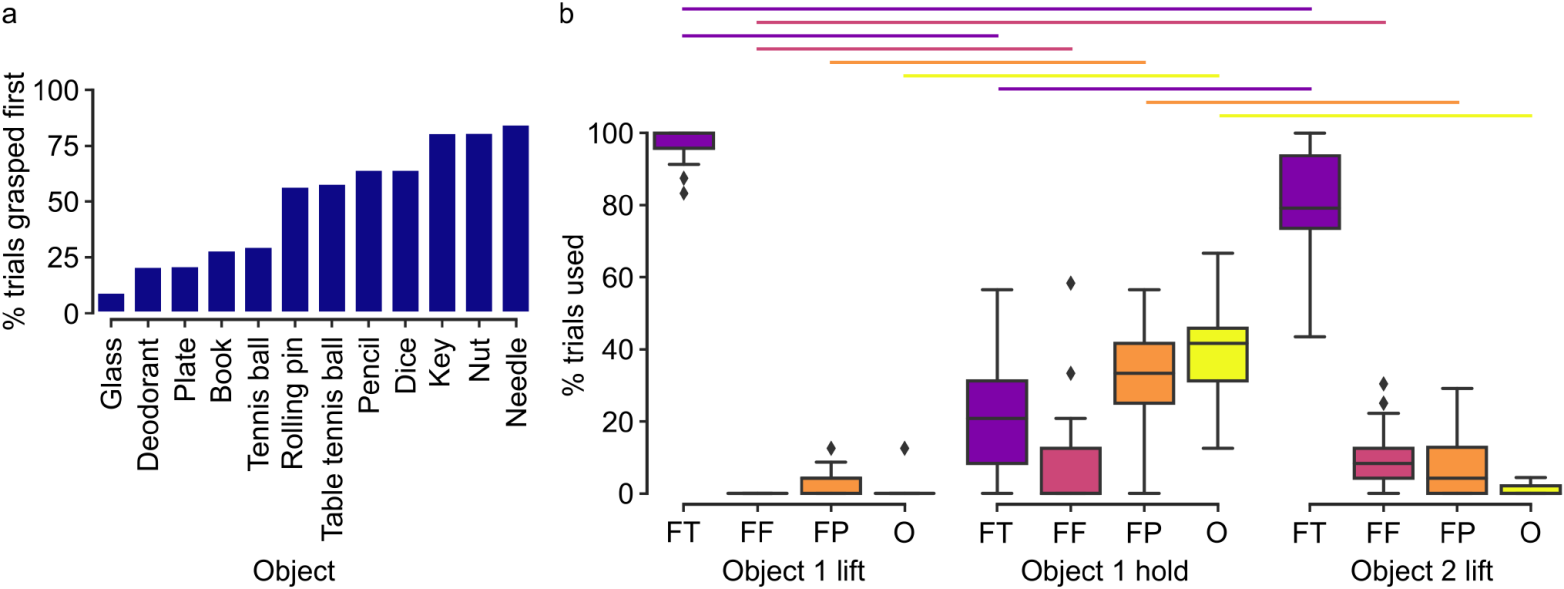
Object order and grip preferences. (**a**) Barplot displaying the percentage of trials in which each object was grasped first. (**b**) Boxplots displaying the percentage of trials in which each grip type was used in each stage (Object 1 lift: grip used for lifting the first object; Object 1 hold: grip used for securing the first object at the point of grasping the second object; Object 2 lift: grip used for lifting the second object). Diamonds indicate outliers beyond 1.5 times the interquartile range. Coloured horizontal lines indicate statistically significant differences between stages for each grip type (*p* < 0.025). FT: *Finger-Thumb*, FF: *Finger-Finger*, FP: *Finger-Palm*, O: *Other*

When examining the percentage of trials (*n* = 639) in which each object was grasped first, we observed that participant preferences appeared to be broadly related to object mass or surface area (i.e., size), with the needle typically chosen first and the glass typically second (Fig. 3a). Across the twelve objects, mass was negatively correlated with the percentage of trials in which the object was grasped first (r_s_(10) = − 0.823, *p* = 0.00100). Across the ten objects for which surface area could be approximated, surface area was also negatively correlated with the percentage of trials in which the object was grasped first (r_s_(8) = − 0.936, *p* < 0.001). Additionally, there was some preference for choosing objects at Position 1 first (mean ± SE = 60.6 ± 2.49% of trials).

### Grip choice

Preferences were also observed in the grip used for each stage of the movement. Multinomial tests indicated that the distribution of grip types was not uniform for Object 1 lift (χ^2^(3) = 1746.35, *p* < 0.001), Object 1 hold (χ^2^(3) = 139.06, *p* < 0.001), and Object 2 lift (χ^2^(3) = 1100.16, *p* < 0.001). *Finger-Thumb* grip was used 96.6% of the time for Object 1 lift (*Finger-Finger*: 0%, *Finger-Palm*: 2.97%, *Other*: 0.469%). Conversely, grip types were more distributed during Object 1 hold, with *Finger-Palm* and *Other* being most frequent (*Finger-Thumb*: 21.1%, *Finger-Finger*: 7.82%, *Finger-Palm*: 33.0%, *Other*: 38.0%). *Finger-Thumb* grip was again more frequently used during Object 2 lift (*Finger-Thumb*: 81.5%, *Finger-Finger*: 10.2%, *Finger-Palm*: 7.20%, *Other*: 1.10%).

Multinomial tests for specific object-order combinations (occurring in over half of the sample) revealed consistency within stages that was not possible to evaluate in the entire sample given the varied object pairings presented to each participant (Supplementary Material, Table S2). When the pencil was Object 1 and book was Object 2 (*n* = 14), 100% of participants used a *Finger-Thumb* grip during Object 1 lift, 92.9% of participants used an *Other* grip during Object 1 hold, and 100% of participants used a *Finger-Thumb* grip for Object 2 lift. A similar pattern of results was observed for rolling pin, plate (*n* = 15) and table tennis ball, plate (*n* = 16), although *Finger-Palm* was the principal grip used for Object 1 hold in these cases. All multinomial tests in this analysis were statistically significant (*p* < 0.001).

When comparing participant grip preferences between stages using a within-subjects analysis, the percentage of trials in which each grip type was used differed (Table 1; Fig. 3b). *Finger-Thumb* grips were significantly more common during Object 1 lift compared to Object 1 hold and Object 2 lift. However, *Finger-Thumb* grips were significantly less common during Object 1 hold compared to Object 2 lift. *Finger-Finger* grips were significantly less common during Object 1 lift compared to Object 1 hold and Object 2 lift. There was no significant difference in percentage for *Finger-Finger* grips between Object 1 hold and Object 2 lift.

**Table 1:** Wilcoxon signed-rank test results

Grip	Mean (SE) %			Object 1 lift versus Object 1 hold			Object 1 lift versus Object 2 lift			Object 1 hold versus Object 2 lift		
	Object 1 lift	Object 1 hold	Object 2 lift	W	*p*	*r*	W	*p*	*r*	W	*p*	*r*
Finger-Thumb	96.6 (0.837)	21.3 (2.93)	81.4 (2.67)	378	< 0.001	1	279.5	< 0.001	0.863	1	< 0.001	− 0.995
Finger-Finger	0 (0)	7.90 (2.53)	10.3 (1.62)	0	0.00163	-1	0	< 0.001	-1	92	0.166	− 0.333
Finger-Palm	2.95 (0.665)	33.1 (2.38)	7.20 (1.61)	0	< 0.001	-1	84	0.101	− 0.391	365	< 0.001	0.929
Other	0.463 (0.463)	37.8 (2.78)	1.09 (0.363)	0	< 0.001	-1	7	0.262	− 0.500	378	< 0.001	1

*Finger-Palm* grips were significantly less common during Object 1 lift compared to Object 1 hold. There was no significant difference in percentage for *Finger-Palm* grips between Object 1 lift and Object 2 lift, but *Finger-Palm* grips were significantly more common during Object 1 hold than Object 2 lift. *Other* grips were significantly less common during Object 1 lift compared to Object 1 hold. There was no significant difference in percentage for *Other* grips between Object 1 lift and Object 2 lift, but *Other* grips were significantly more common during Object 1 hold compared to Object 2 lift.

Visualising grip choices in the context of object combinations revealed further detail. As already shown in Fig. 3b, Object 1 and Object 2 were frequently grasped with a *Finger-Thumb* grip (Supplementary Material, Fig. S1 and Fig. S2), with the tennis ball being the only notable deviation for Object 1 lift, where a *Finger-Palm* grip was occasionally used (Supplementary Material, Fig. S1). There was some use of *Finger-Finger* or *Finger-Palm* grip for Object 2 lift when Object 1 was smaller or lower mass (key, nut, needle; Supplementary Material, Fig. S2).

**Fig. 4:**
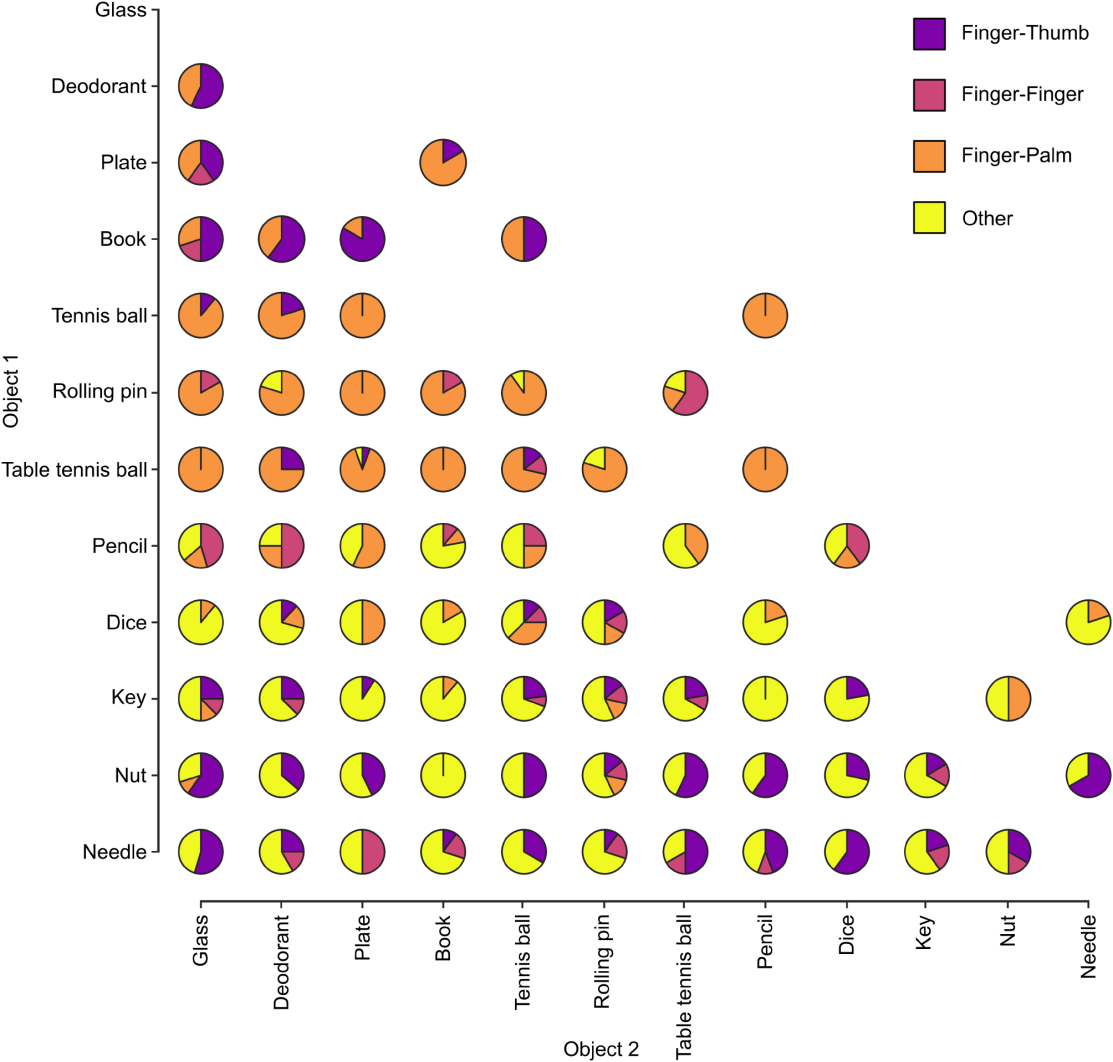
Object interactions for grip type during Object 1 hold. Pie charts represent the proportion of trials in which each grip type was used for holding Object 1 prior to grasping Object 2. For example, when the table tennis ball was chosen as Object 1, it was held using a *Finger-Palm* grip in 100% of trials if Object 2 was the glass or the book, but 75% of trials if Object 2 was the deodorant (with a *Finger-Thumb* grip used for the remaining 25% of trials). Objects are ordered according to selection preference shown in Fig. 3a. Note that some object combinations are absent, either because they never occurred (e.g., deodorant was never selected as Object 1 with needle as Object 2, only ever the opposite) or because they occurred in less than 4 trials

There was greater variability during Object 1 hold (Fig. 4) than for Object 1 lift and Object 2 lift. A clear distribution of grip types was observed when sorting based on object order preference (as in Fig. 3a). Notably, objects with a larger mass or higher surface area (deodorant, plate, book, tennis ball, rolling pin) as well as the table tennis ball were preferentially held using a *Finger-Thumb* or *Finger-Palm* grip prior to grasping Object 2. *Finger-Thumb* grips were more common in the rare instances that Object 1 was the deodorant or book. Objects with the smallest mass or lowest surface area (pencil, dice, key, nut, needle) often elicited a *Finger-Thumb* or *Other* grip during Object 1 hold.

## Discussion

In this exploratory work we examined the object selection preferences and grip types used during multiple object handling with pairs of familiar objects. Participants were presented with the object pairs and grasped them in an order of their choosing. We calculated the frequency each object was grasped first and how this was related to object mass and surface area. We also coded typical (finger-thumb opposition) and atypical grip types and examined how these were distributed within and between three stages of the action (Object 1 lift, Object 1 hold, Object 2 lift). We visualised grip choice within object pairs for a descriptive analysis. Results indicated that finger-thumb opposition was the most prevalent grip type for lifting objects, whilst a range of atypical grips were used for holding Object 1. In addition, object mass or surface area were correlated with selection order, such that smaller objects were more often selected first. Below we discuss these results in more detail, consider the use of atypical grips in motor control, address limitations of our study, and propose potential areas for future research.

### A possible common approach to multiple object handling

The pattern of grip types used within and between stages provides preliminary evidence for an overarching approach to multiple object handling (at least for situations involving two objects). Specifically, we observed that when two objects are grasped cumulatively, finger-thumb opposition is almost always used to grasp the first object (as is typically observed for single-object interactions). The first object is then held within the hand, frequently using an atypical grip. This is probably to ensure that finger-thumb opposition can be used again for grasping a second object, where this grip type is once again the most common. Importantly, this general strategy was clearly evident when examining the specific object-order combinations that occurred in at least half of participants.

Post hoc analysis indicated a negative correlation between the mass or surface area of each object and the percentage of trials in which it was selected first. Given the general strategy described above, it is plausible that individuals select an object that is lighter or with lower surface area first to increase the ease of holding this object during the second grasp and subsequent transport. Whilst the correlations should be considered with some caution given the low number of datapoints, this explanation is highly feasible given that it is much easier to grasp a large (heavy) object when a small (light) object is already in the hand than vice versa. Interestingly, we did find that there was some preference for participants to select the object closest to them to grasp first. However, this is unsurprising given that half of the objects had a mass below 10 g (table tennis ball, pencil, dice, key, nut, needle), three between 30 and 80 g (deodorant, tennis ball, rolling pin), and three greater than 100 g (glass, plate, book). Given the strategy described above it is likely that when objects of similar mass (or size) are paired one can select them in either order (and so participants grasp the nearest object first).

Our results highlight the fact that multiple object handling is a dexterous motor skill that is both conservative and flexible. It is conservative in that finger-thumb opposition appears to be prioritised for grasping where possible, maintaining this effective approach to grip objects in a secure manner. This is particularly evident when examining the lifting of the first object, where participants almost never used an atypical grip. Multiple object handling is also flexible in that it elicits a range of atypical grips to hold, and occasionally grasp, objects. Indeed, atypical grips were occasionally used to lift a second object, deviating from the common approach described above. One particularly notable example was the glass, in which finger-finger opposition was occasionally used (Supplementary Material, Fig. S2). That the hollow structure of a drinking glass promotes lifting between the fingers is unsurprising given that this is a highly familiar action. However, as visualised in Fig. S2, preferences for lifting using an atypical grip are also linked to a (limited) variety of other objects and object combinations. It will now be beneficial to move beyond purely descriptive visualisation and develop a deeper understanding of how different objects (and their properties) interact to elicit certain grip types.

### Atypical grips in multiple object handling

In highlighting the use of atypical grips in multiple object handling, the present work extends our understanding of the capacity of the human hand for dexterous motor control. We observed that participants were able to effectively manipulate the first object within the hand and then hold it securely using one of a range of atypical grips, thus facilitating cumulative object grasping and subsequent transport. As far as we are aware, these atypical grips are not yet captured in grasp taxonomies that focus on single object interactions (although, of course, they are more commonly used for holding than grasping in the present work). Notably, atypical grip choice may be linked to object size in much the same way that typical power and precision grips are (Castiello 2005). In addition to this, object properties and action stage also appear to interact to influence grip choice, in a way that is unique to multiple object handling. For example, some objects (tennis ball, rolling pin, table tennis ball) were commonly held between the fingers and the palm after grasping when they were selected as the first object (Fig. 4). Conversely, objects with the smallest mass or lowest surface area (pencil, dice, key, nut, needle) were frequently held after grasping using the *Other* grip category, which involved a variety of potential grips (e.g., fingers opposing themselves in a ‘hooked’ posture). As mentioned above, these results are purely descriptive, but they do allow the visualisation of patterns across the stimulus set and facilitate the creation of new research questions regarding object shape and size that will be beneficial to evaluate in future work (for example, whether there is an object size at which finger-palm opposition becomes preferred for holding).

Unfortunately, when developing the coding scheme, we did not appreciate the extent to which the *Other* category would be used in the full dataset. In fact, we observed several atypical grip types in the full sample that were suitable for the *Other* category but not seen in the participants with which the coding scheme was developed. Our present results are therefore lacking a fine-grained evaluation of the range of grips falling into this category. Further study will be necessary to evaluate the prevalence of the different grips captured in the *Other* category (e.g., thumb-palm opposition, use of the palmar creases) and how they might be linked to object properties. Indeed, in addition to widening the categorisation of atypical grip, more work will be necessary to further understand other aspects of multiple object handling (see below).

### Limitations

We must consider potential limitations of our study and caveats to the interpretation of results provided above. As a preliminary investigation into multiple object handling, we sought to study it in a semi-naturalistic fashion by using familiar objects which, if grasped individually, afforded an extensive variety of potential finger-thumb opposition grips (thus accounting for established variability in the grip types that occur in typical, everyday object grasping). Consequently, the properties of the objects were not distributed in a controlled manner. Given that object order preference was closely related to object mass or surface area, these features formed the basis for interpreting our results. Whilst all objects differed in other properties (e.g., shape, texture), these were not evaluated in our analysis. Further work using custom experimental stimuli, such that object properties and their combinations are controlled and evenly distributed, would allow one to more comprehensively examine the links between action stage, grip type, and object properties.

In addition to focussing on familiar objects, we also decided against testing participants on all possible object combinations, given the extended experiment duration this would have entailed. Furthermore, to evaluate object selection preferences, we allowed participants to choose the order in which they grasped objects. As stated in the method section, these two factors mean that the percentage use of each grip type was associated with different object pairs for each participant. For example, some participants could have grasped the key as Object 1 and the dice as Object 2, some may have grasped these objects in the opposite order, and others may not have been presented with this combination at all. As such, the distribution of grips can vary due to participants interacting with different objects, and not because they used different grips for a matched set of objects. Regardless, the prevalence of finger-thumb opposition during Object 1 and Object 2 lift is likely to reflect an invariant approach that is not confined to a specific object (and this was supported by multinomial analyses of three participant subsamples with matched object-order combinations).

One notable constraint of our work is the lack of a formally validated coding scheme. Whilst there was a high level of agreement between the first coder and the subsequent verification by another author, coding was not performed in a truly independent manner (i.e., with two or more raters coding the same behaviour individually and then calculating inter-rater reliability). Although we are confident that the overarching strategy we describe cannot be explained by biased coding (i.e., through erroneously coding atypical grips as finger-thumb opposition), ensuring the reliability of coding should be a priority in future research on multiple object handling. Another potential limitation is the absence of detailed participant information. For example, we did not collect relevant data to allow us to exclude participants who had a motor disorder or expertise in a dexterous skill (e.g., instrument playing, sports). These factors may have reduced or improved participant ability in completing the experimental task. However, we did not observe that any participant had specific difficulties completing the task that would indicate problems with dexterous motor control. We also do not consider enhanced manual dexterity a particular concern, since we consider it unlikely that prioritising finger-thumb opposition (the grip type normally used for grasping single objects) would be a strategy used only by those with enhanced manual dexterity. One could argue that individual differences in manual dexterity might explain grip type outliers (Fig. 3b). However, these are also plausibly explained by the unique object pairs those participants interacted with. Future examination of individual differences in multiple object handling might help us understand whether this efficient method for object transport is negatively impacted by certain clinical disorders and whether (and why) some individuals might be more skilled in this behaviour than others.

A final limitation is the potential for experimenter bias in guiding participant responses. That is, since participants were provided with a demonstration by the experimenters and were able to observe them manipulate objects between trials it is possible that this could explain the prevalence of the general approach to multiple object handling proposed above. Whilst we cannot fully exclude this possibility, we consider it unlikely. Participants were only ever provided a formal demonstration with the first object pair (one of 66 possible combinations). We did not control for the strategy used during demonstration or experimenter behaviour between trials, but we made it clear to participants that they could complete the task in whichever manner they saw fit, as long as they did not let the first object touch the table after lifting it or use one object to carry another.

Ultimately, given that participants had no problem using a wide variety of different grips in the Object 1 hold stage, we think it is unlikely that they would have chosen to mainly use finger-thumb opposition for grasping Object 1 and Object 2 solely due to experimenter guidance if there was an alternative strategy that they found easier. The overarching approach to multiple object handling described above was incredibly consistent across participants (regardless of the different object pairs they interacted with) and is highly plausible a priori: it is clearly evident if one engages in multiple object handling that finger-thumb opposition is an effective way to grasp at least the first object. In any case, the current work provides a basis for further examination of multiple object handling in a more controlled manner to verify the results reported here.

### Future research

The current work provides a first step towards a deeper understanding of multiple object handling, and so there is considerable potential for further research in addition to that mentioned above. It is worth emphasising that whilst the preferential use of finger-thumb opposition for grasping and atypical grips for holding is likely reflected in naturalistic behaviour, the distribution of grip types we observed within each stage is unlikely to be indicative of their prevalence of use outside of a laboratory environment (and our study was not designed to evaluate this). Rather, the present results are more informative regarding the types of grip that *can* be used during multiple object handling, and how the grips used might vary between stages (particularly when one grip type, finger-thumb opposition, is highly skewed). Similarly, given that the objects used in this experiment were chosen primarily for their capacity to elicit varied (finger-thumb opposition) grips, their properties may not necessarily reflect those of objects people naturally choose to transport through multiple object handling. Recording freely-acting participants as they complete their daily activities would reveal more about grip prevalence, the scenarios in which certain grips occur, and the objects that are typically involved in multiple object handling (Bullock et al. 2013; Ingram et al. 2008; Nakamura et al. 2017).

It would also be beneficial to examine multiple object handling with more than two objects (either in a laboratory or naturalistic environment), since we are not limited to this number in our daily activities. We assume that the general approach described above would remain, with finger-thumb opposition being prioritised for each grasp and each prior object commonly held using an atypical grip. Naturally, this will become more challenging with each additional object as the capacity of the hand is reduced and the cumulative object property interactions become more complex. Additionally, it is well-established that the kinematics of reaching and grasping are influenced by object properties and grip (e.g., Castiello et al. 1993; Gentilucci et al. 1991; Marteniuk et al. 1990). Examining the kinematics of multiple object handling would extend our understanding of how such actions are planned and performed. For example, by showing how the spatial and temporal features of the reach-to-grasp action change depending on the requirement for a subsequent grasp and the grip used for holding.

Further study of multiple object handling is likely to have benefits beyond developing our understanding of human motor control. Specifically, such investigation may be informative for the design of advanced robotics. The study of human hand control has long been influential on robotics development, but robots cannot yet fully replicate the dexterity of the hand (Billard and Kragic 2019). Some researchers have begun to develop robots that can engage in multiple object handling in a human-like fashion (Yao and Billard 2023), but there has been a stronger focus on the grasping of multiple objects simultaneously rather than cumulatively (Li et al. 2024; Sun et al. 2022). This skill, which has been referred to as ‘multi-object grasping’ (Sun et al. 2022), is also commonly used by humans for efficient object transport (e.g., in cases where one might want to grasp several small items of food from a bowl at once). However, it is distinct from multiple object handling and poses its own unique challenges (both for the human sensorimotor system and robots).

### Conclusion

To conclude, the present work provides insight into the strategies used for multiple object handling, a common skill for transporting several objects simultaneously. Results suggest that the overarching approach to this task is to use finger-thumb opposition where possible, alongside a variety of atypical grips to facilitate the holding of a previously grasped object. These findings provide the basis for further research into this unique motor skill and contribute to our broader understanding of human manual dexterity.

## Electronic supplementary material

Below is the link to the electronic supplementary material.

Supplementary Material 1

## Data Availability

Processed data are freely available on the Open Science Framework: 10.17605/OSF.IO/4E7DN.
