# Supplementary material for: Multiple object handling: exploring strategies for cumulative grasping and transport using a single hand
Source: Exp Brain Res. 2025 Jun 3;243(7):165. doi: 10.1007/s00221-025-07084-x (PMC12134043; doi:10.1007/s00221-025-07084-x)
Supplement: Supplementary file 1 — Supplementary Material 1 [file 221_2025_7084_MOESM1_ESM.pdf]

Multiple object handling: exploring strategies for cumulative grasping and transport using a single hand

Arran T. Reader*, Laura Gaile, Wenxi Li, Emily E. Cheah Mc Corry, Kirsten Mackie

Department of Psychology, Faculty of Natural Sciences, University of Stirling, Stirling, UK

* Corresponding author: arran.reader@stir.ac.uk

Table S1: Object alignment with grasp taxonomies

Objects were chosen such that a variety of grip types could be elicited, aligned with published grasp taxonomies. Only the grips categorised by Feix et al. (2016) that were not also present in Stival et al. (2019) are listed. Note that some grips can be used for multiple objects.

Taxonomy	Grip type		Included?	Example object
Stival et al. (2019)	1. Flat grasps	Lateral	Yes	Key
		Extension type	Yes	Plate
		Quadpod	Yes	Table tennis ball
		Parallel extension	Yes	Book
	Index finger extension		No (tool-specific)	n/a
	2. Distal grasps	Stick	Yes	Pencil
		Writing tripod	Yes	Pencil
		Prismatic four fingers	Yes	Pencil
		Power disk	Yes	Plate
	3. Cylindrical grasps	Large diameter	No (uses entire hand)	n/a
		Medium wrap	Yes	Deodorant
		Small diameter		Rolling pin
		Fixed hook	Yes	Glass
	4. Spherical grasps	Tripod	Yes	Table Tennis ball
		Power sphere	Yes	Tennis ball
		Precision sphere	Yes	Tennis ball
	5. Ring grasps	Three fingers sphere	Yes	Tennis ball
		Prismatic pinch	Yes	Nut
		Tip pinch	Yes	Needle
		Ring	Yes	Deodorant
Feix et al. (2016)	Adducted thumb		Yes	Deodorant
	Light tool		Yes	Pencil
	Palmar		Yes	Plate
	Sphere 4 fingers		Yes	Tennis ball
	Distal type		No (tool-specific)	n/a
	Adduction grip		Yes	Pencil
	Ventral		Yes	Pencil
	Lateral tripod		Yes	Dice
	Tripod variation		No (tool-specific)	n/a
	Inferior pincer		Yes	Table tennis ball
	Prismatic 2 fingers		Yes	Pencil
	Prismatic 3 fingers		Yes	Pencil
	Precision disk		Yes	Plate

Table S2: Multinomial tests for matched object-order combinations

All tests statistically significant, p < .001.

Object 1	Object 2	n	Percentage grip type												Multinomial test, $\chi^2(3)$		
			Object 1 lift				Object 1 hold				Object 2 lift				Object 1 lift	Object 1 hold	Object 2 lift
			FT	FF	FP	O	FT	FF	FP	O	FT	FF	FP	O			
Pencil	Book	14	100	0	0	0	0	7.14	0	92.86	100	0	0	0	42	34.6	42
Rolling pin	Plate	15	100	0	0	0	0	0	100	0	100	0	0	0	45	45	45
Table tennis ball	Plate	16	87.5	0	12.5	0	6.25	0	87.5	6.25	93.75	6.25	0	0	34	33.5	40.5

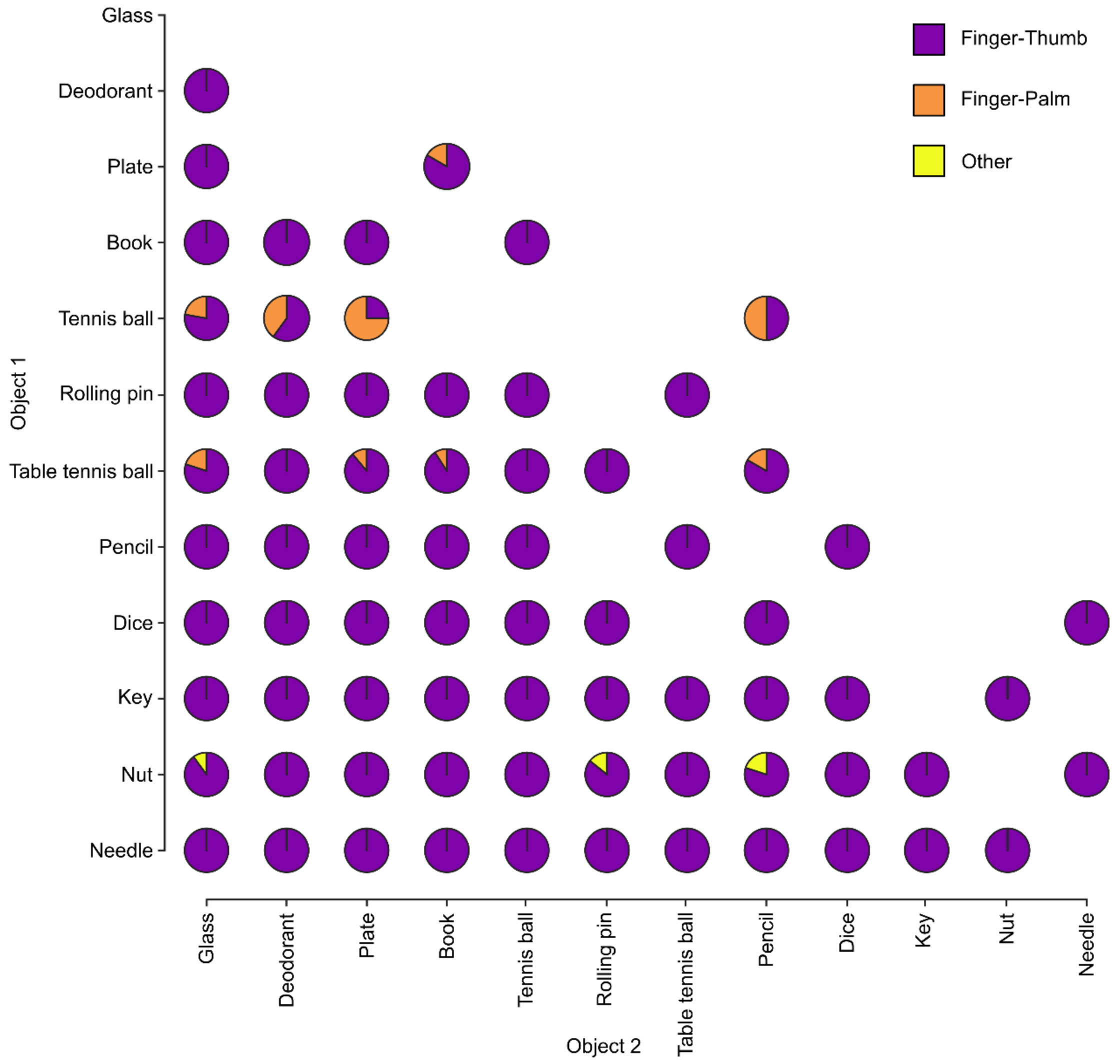

Fig. S1: Object interactions for grip type during Object 1 lift

Pie charts represent the proportion of trials in which each grip type was used for lifting Object 1. For example, when the tennis ball was chosen as Object 1 and Object 2 was the plate, it was grasped using a *Finger-Palm* grip in 75% of trials and *Finger-Thumb* grip in 25% of trials. Objects are ordered according to selection preference shown in Fig. 3a. Note that some combinations are absent, either because they never occurred or because they occurred in less than 4 trials.

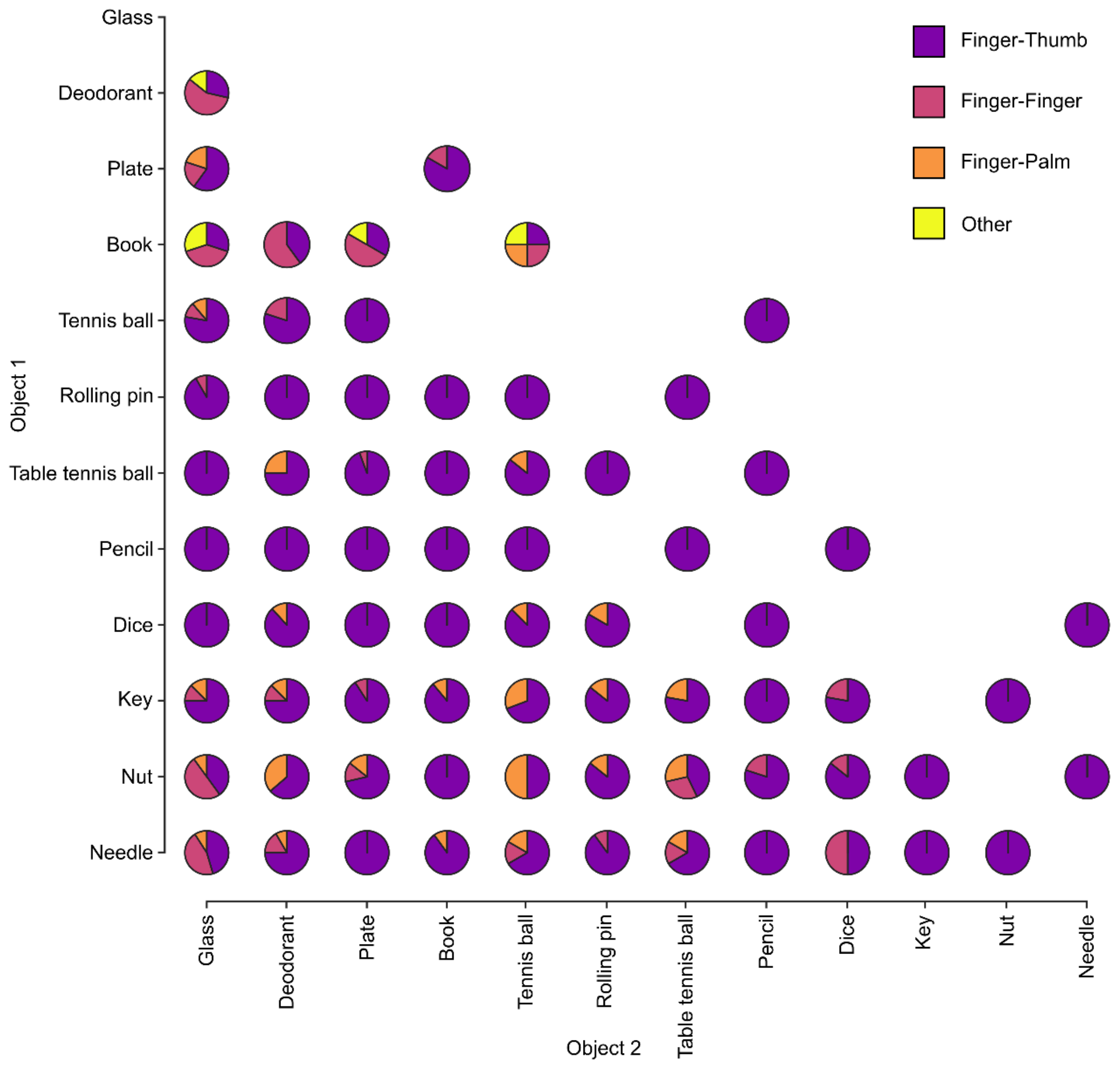

Fig. S2: Object interactions for grip type during Object 2 lift

Pie charts represent the proportion of trials in which each grip type was used for lifting Object 2. For example, when the tennis ball was chosen as Object 2 and Object 1 was the nut, the tennis ball was grasped using a *Finger-Palm* grip in 50% of trials and a *Finger-Thumb* grip in the other 50% of trials. Objects are ordered according to selection preference shown in Fig. 3a. Note that some combinations are absent, either because they never occurred or because they occurred in less than 4 trials.
